# Intravascular Ultrasound Guided Intervention in Calcified Coronary Lesions Showed Good Clinical Outcomes during One Year Follow-Up

**DOI:** 10.3390/jcm12124073

**Published:** 2023-06-15

**Authors:** Khanh-Hung Doan, Tai-Li Liu, Won-Sik Yun, Yi-Sik Kim, Kyeong Ho Yun, Seok Kyu Oh, Jong-Pil Park, Jay Young Rhew, Sang-Rok Lee

**Affiliations:** 1 Division of Cardiology, Jeonbuk National University Hospital, Jeonju 54907, Republic of Korea; 2 Division of Cardiology, Wonkwang University Hospital, Iksan 54538, Republic of Korea; 3 Division of Cardiology, Presbyterian Medical Center, Jeonju 54987, Republic of Korea; 4 Research Institute of Clinical Medicine, Jeonbuk National University Hospital, Jeonbuk National University Medical School, Jeonju 54907, Republic of Korea

**Keywords:** percutaneous coronary intervention, drug-eluting stents, calcium, intravascular ultrasound

## Abstract

Background: Calcified coronary lesions can cause stent under-expansion, malapposition, and polymer degradation, hence increasing the risk of adverse clinical outcomes. Percutaneous coronary intervention (PCI) guided by intravascular ultrasound (IVUS) has been used regularly to improve outcomes. Our primary aim was to evaluate the clinical efficacy of IVUS-guided PCI in calcified coronary lesions. Methods: From August 2018 to December 2021, we prospectively included 300 patients in the CAPIRO study (CAlcified plaque in patients receiving Resolute Onyx^®^) at three educational hospitals in Jeonbuk Province. We studied 243 patients (265 lesions) who were followed up for over a year. Based on coronary calcification by IVUS analysis, the patient population was categorized into two groups (Group I: non/mild calcification; Group II: moderate/severe calcification (maximum calcium arc >180° and calcium length > 5 mm)). One-to-one Propensity Score Matching was used to match the baseline characteristics. The stent expansion rate was analyzed by recent criteria. The primary clinical outcome was Major Adverse Cardiac Events (MACE), which included Cardiac death, Myocardial Infarction (MI), and Target Lesion Revascularization (TLR). Results: After follow-up time, the MACE rate in Group I was 1.99%, comparable to Group II’s 1.09% (*p* = 0.594). The components of MACE did not significantly differ between the two groups. Based on absolute MSA or MSA/MVA at MSA site criteria, the stent expansion rate in Group II was lower than that of Group I. Nevertheless, based on recent relative criteria, the stent expansion rate in both groups was comparable. Conclusions: After more than a year of follow-up, IVUS-guided PCI in moderate/severe calcification lesions was associated with good clinical outcomes, which was comparable with non/mild calcification lesions. Future studies with a larger sample size and a more extended follow-up period are required to clarify our findings.

## 1. Introduction

Although ongoing technological advancements in drug-eluting stents (DES) and procedural techniques increasingly allow Percutaneous Coronary Intervention (PCI) for more high-risk and complex coronary lesions, there is still a sizable percentage of patients who experience stent-related major adverse cardiovascular events (MACE) following PCI [1]. A calcified plaque, extreme tortuosity, a large thrombus burden, and diffuse lesions with poor stent landing zones were all related to worse outcomes [2]. Lesions with a high calcification burden pose the most significant challenge and are most likely to have an adverse effect on PCI outcomes. Severe coronary calcification is independently associated with increased rates of MACE because calcified target lesions may result in reduced minimum stent area, stent under-expansion, stent malapposition, polymer degradation, and greater risks of restenosis and stent thrombosis [3,4,5,6,7,8].

In addition to coronary angiography, PCI procedures are increasingly being facilitated by intracoronary imaging modalities such as intravascular ultrasound (IVUS) and optical coherence tomography (OCT). IVUS and OCT offer more accurate assessments of stenosis degree, lesion length, plaque burden, degree of calcification, and plaque features when compared to coronary angiography only [9]. Using IVUS during PCI did not increase the cost-effectiveness rate of the procedure [10]. For the calcification lesions, OCT and IVUS could measure calcium arc, clusters of microcalcifications, or densely calcified plaque, and OCT could measure calcium thickness [2,11,12]. This information could direct a strategy for lesion preparation before stenting, such as balloon angioplasty, mechanical atherectomy, laser atherectomy, or lithoplasty, and it could also optimize stent implantation, such as expansion rate, apposition, and complications [2,11,13,14,15,16,17]. According to certain studies, IVUS-guided PCI may produce better results than angiography guidance, especially in patients with complex coronary artery lesions [18,19,20], and suggest some criteria for optimal stent expansion [21,22,23,24,25].

However, the safety and effectiveness of IVUS-guided PCI in patients with calcified plaques are still uncertain. The CAPIRO (CAlcified Plaque in Patients Receiving Resolute Onyx^®^) study’s objective was to evaluate the role of IVUS-guided PCI in calcified coronary lesions and the clinical outcomes after at least one year of follow-up.

## 2. Materials and Methods

### 2.1. Study Designs

CAPIRO is a prospective, multi-center study designed to assess the impact of IVUS-guided PCI in patients with calcified lesions who received a Zotarolimus-Eluting Coronary Stent (Resolute Onyx^TM^ DES, Medtronic, Minneapolis, MN, USA). From August 2018 to December 2021, the study was conducted in three educational hospitals in Jeonbuk Province, Korea (Jeonbuk National University Hospital, Wonkwang University Hospital, and Jeonju Presbyterian Medical Center). Each hospital included in the study approved the study protocol. All enrolled patients received the explanation of the study and signed an informed consent form. This study evaluated 243 patients (with 265 lesions) who were followed up for more than one year. The patient population was separated into two groups based on the degree of calcification detected by IVUS. In our study, moderate or severe calcification was categorized as lesions with a maximum calcium arc >180° and a calcium length of more than 5 mm (Group II: 92 patients, 94 lesions); otherwise, lesions were classified as non or mild calcification (Group I: 151 patients, 171 lesions). If a patient has numerous lesions, classification is based on the lesion with the most significant calcification.

**Figure 1:**
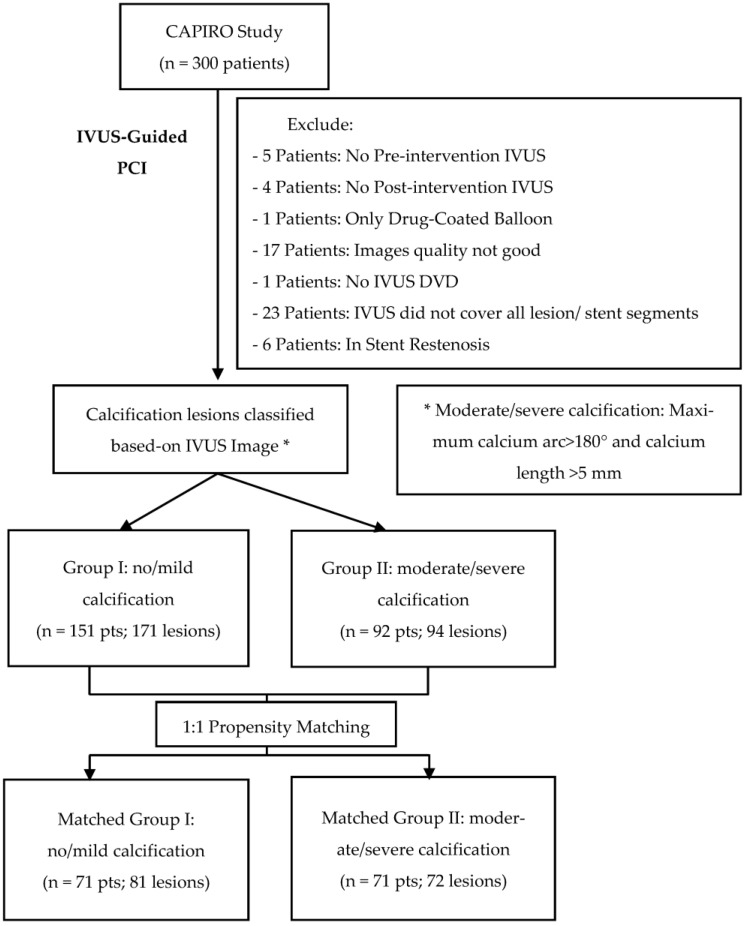
Study Flowchart.

Propensity score matching (PSM) was performed for age, male gender, history of diabetes mellitus, chronic kidney disease, current smoking, and LVEF with matching tolerance of 0.2. After the PSM, the patient population was categorized into matched-group I (n = 71 patients, 81 lesions) and matched-group II (n = 71 patients, 72 lesions) (Figure 1).

### 2.2. Study Criteria

Inclusion Criteria:

- 18 years old or older.
- Patients with coronary artery disease who meet the criteria for index PCI according to current recommendations for myocardial revascularization [26,27].
- Patients consented to participate and signed informed consent forms.
- Exclusion Criteria:
- Cardiogenic shock.
- Contraindications for dual antiplatelet therapy for 12 months.
- Major bleeding within three months or major surgery within two months.
- Life expectancy is less than one year.
- In-stent restenosis lesion.
- IVUS image-related exclusions: lack of pre- or post-intervention IVUS, lack of coverage of all lesion or stent segments, and insufficient image quality for analysis.

### 2.3. IVUS-Guided PCI Procedure

All PCI procedures were carried out following current technological standards and guidelines [26,27]. All patients were administered a loading dose of antiplatelet medications (300 mg Aspirin and 600 mg Clopidogrel or 180 mg Ticagrelor) before the procedure. After obtaining vascular access via the radial or femoral artery, a loading dose of 70–100 IU/kg of Unfractionated Heparin was administered to achieve an Activated clotting time (ACT) of 250–300 seconds. If the procedure duration exceeds one hour, an extra 3000 IU of Unfractionated Heparin is administered, or the ACT findings are used to determine the amount of additional Unfractionated Heparin.

Our study utilized commercially available IVUS systems, including the Boston Scientific POLARIS Multi-Modality Guidance System with OPTICROSS^TM^ and OPTICROSS^TM^ HD Coronary Imaging Catheter and the Philips Volcano IVUS System with Eagle Eye Platinum Catheter. Lesions with total occlusion or severe stenosis that the IVUS catheter may not be able to cross were pre-dilated with a compliant balloon. After administering 100–200 ug of intracoronary Nitroglycerin, the IVUS catheter was advanced through the target lesion to a distance of at least 10 mm distal. The IVUS catheter was automatically pulled back at a rate of 0.5 mm/s for measurements on-site. On-site IVUS measures before stent implantation comprised minimal lumen diameter, minimal lumen area (MLA), reference lumen area, plaque burden, and lesion morphology (calcified, lipid-rich plaque). Utilization of a non-compliant balloon or adjunctive lesion preparation was left to the clinician’s discretion. The stent’s diameter, size, and landing zone were selected based on recent consensus documents [28]. In our study, every patient received the Zotarolimus-Eluting Coronary Stent system (Resolute Onyx^TM^ DES, Medtronic). After stent implantation, IVUS images were acquired to evaluate stent deployment results (minimum stent area (MSA), expansion rate, apposition) and acute complications (dissection, thrombus, hematoma, and tissue protrusion). Using an additional non-compliant balloon for post-dilation was determined by clinical circumstances and the clinician’s discretion. The final IVUS image was recorded. All IVUS images were saved to DVD for offline analysis.

After PCI, patients were treated with guideline-directed medical therapy. This therapy included dual-antiplatelet therapy for 12 months, beta-blockers, statins, and other medications based on the patient’s specific clinical circumstances.

### 2.4. IVUS Images Analysis

Based on recent consensus documents, IVUS images were analyzed offline every 1 mm using planimetry software QIvus Research Edition 3.1. (Medis Medical Imaging System B.V., Leiden, The Netherlands) [29,30]. The IVUS image analyzers were blinded from the patient’s clinical information. The evaluated segments contained the target lesion (pre-PCI) or stent segment (post-PCI), as well as 5 mm proximal and distal reference segments with no intervening branches. Pre-PCI parameters include minimal luminal area, plaque burden at minimal luminal area site, proximal, distal, mean luminal reference area and plaque burden, remodeling index, and lesion length. The pre-PCI lesion morphology analysis includes calcium morphology, calcium nodule, maximal calcium arc, total calcium length, calcium index ((maximum calcium arc/360) × (calcium length/lesion length) × 100), attenuated plaque, and plaque rupture. Following the PCI, at the minimal stent area site, stent area (MSA), maximal stent diameter, minimal stent diameter, stent asymmetry, stent eccentricity, and stent length were examined. Stent expansion was assessed by absolute MSA, Conventional stent expansion (MSA/mean reference luminal area × 100%) [28], MSA/MVA at MSA site [25], IVUS-XPL (MSA > distal reference luminal area) [23], ULTIMATE criteria (MSA > 5.5 mm^2^ or >90% distal lumen reference area) [21,22]. Complications of PCI procedures such as stent edge dissection, malapposition, and tissue protrusion were also evaluated. Stent edge dissection was considered significant if the dissection flap opened more than 60°, if it reached the media, or if it was longer than 2 mm. Malapposition was considered major when the axial distance was >0.4 mm or >1 mm in length [28].

### 2.5. Quantitative Coronary Angiography (QCA)

Coronary angiography images from the intervention procedure were archived and evaluated by a different physician who was uninformed of IVUS images. QAngio XA 7.3.102.0 (Medis Medical Imaging System B.V., The Netherlands) was utilized for the QCA analysis. Using the catheter size for calibration was the first stage of the QCA process. Following this, a vessel line was drawn with 5 mm proximal and distal margins on both sides of the target lesion. The vessel contour was then automatically generated. The margin of the lesion, as well as the proximal or distal segment, was determined manually. From the QCA, it was possible to extract the following parameters: lesion diameter, lesion reference diameter, diameter stenosis, lesion area, lesion reference area, proximal reference diameter and area, distal reference diameter and area, plaque symmetry, and plaque area. In the case of total occlusion, the lesion length would be unavailable, and the reference diameter would be the diameter of the proximal reference segment [31].

### 2.6. Follow-Up and Endpoints

All patients should return for follow-up visits to the outpatient clinic after one, six, 12 months, and every six months; in some cases, patients were also contacted by phone at these intervals. The primary endpoint of the study is Major Adverse Cardiac Events (MACE) after the follow-up time, which is defined as the composite of Cardiac death, Myocardial Infarction (MI) of the target vessel, and Ischemia-driven Target lesion revascularization (TLR). If cardiac-related causes of death are discovered, a diagnosis of cardiac death is made. In all other cases, death was classed as non-cardiac death. Myocardial Infarction was defined according to the current definition of myocardial infarction [32]. Ichemia-driven target lesion revascularization consists of revascularization within the target lesion and 5 mm proximal and distal to the target lesion. Secondary endpoints include Patient-oriented composite endpoint (all-cause death, MI, and any revascularization), the individual components of MACE, all-cause death, stroke, target vessel revascularization (TVR), other vessel revascularization (OVR), chronic heart failure (CHF), and Stent thrombosis.

### 2.7. Statistical Analysis

All continuous variables were reported as means ± standard deviations or as median (interquartile range) and compared using Student’s t-test or Mann–Whitney U test, as appropriate. The Chi-square test or Fisher’s exact test, as appropriate, was used to compare categorical variables, represented as percentages (numbers). The baseline characteristics of both groups were matched by using Propensity score matching on a one-to-one basis. Before matching, age, male gender, diabetes mellitus history, chronic kidney disease, current smoking, and LVEF were established as predictors for the difference between the two groups. The tolerance for matching was set to 0.2. After at least one year of follow-up, the clinical outcomes were examined using the Kaplan–Meier method, and the difference between the two groups was determined using the log-rank test. All statistical analyses were conducted using version 26 of IBM SPSS Statistics (IBM Corporation, Armonk, NY, USA). Two-sided *p* values below 0.05 were regarded as statistically significant.

## 3. Results

### 3.1. Baseline Clinical Characteristics

Table 1 compares the baseline clinical characteristics of both groups before and after the PSM. Before the PSM, patients in Group II were older than those in Group I (mean age 67.71 ± 9.77 vs. 63.60 ± 9.75, *p* = 0.002). The proportion of patients over 65 years old in Group II was higher (61.96%, as opposed to 40.40% in Group I, *p* = 0.001). Group II also has more patients with diabetes mellitus (53.26% vs. 24.50%, *p* = 0.001), chronic kidney disease (8.70% vs. 1.32%, *p* = 0.005), and fewer patients who are male (65.22% vs. 82.12%, *p* = 0.003) and currently smoke (14.13% vs. 29.14%, *p* = 0.035). According to the findings of the common laboratory tests, patients in Group I have greater hemoglobin, EGFR, and LVEF values than patients in Group II. After the PSM, all clinical characteristics were comparable between the two groups, except that HbA1c was higher in Group II (6.15% (5.70,7.43) vs. 5.80% (5.50,6.30), *p* = 0.011) and there was a tendency for Group II to have more patients with diabetes mellitus (45.07% in Group II vs. 29.58% in Group I, *p* = 0.056).

### 3.2. Coronary Angiography Analysis

Before the PSM, Table 2 showed that most of the lesions in the two groups were in the LAD artery (68.42% in Group I and 70.21% in Group II, *p* = 0.763). Group II had a more significant percentage of lesions classified as type C according to the AHA/ACC classification (54.26% vs. 35.67%, *p* = 0.003). Group I had significantly more lesions with TIMI-2 (21.05% vs. 10.64%, *p* = 0.032) and significantly fewer lesions with TIMI-3 than Group II (63.16% vs. 76.60%, *p* = 0.025). Based on QCA analysis, the lesion length was longer in Group II compared to Group I (27.30 ± 9.94 vs. 23.95 ± 8.56, *p* = 0.005). However, compared to Group II, Group I has a greater lesion reference diameter (2.94 ± 0.59 vs. 2.67 ± 0.63, *p* = 0.001), proximal mean diameter (3.28 ± 0.56 vs. 3.04 ± 0.60, *p* = 0.002), and distal mean diameter (2.92 ± 0.64 vs. 2.75 ± 0.79, *p* = 0.047). Plaque symmetry and plaque area were comparable between the two groups. After the PSM, all QCA analysis parameters were equal across the two groups, except for the lesion reference diameter, which remained significantly smaller in Group II (2.70 ± 0.63 vs. 2.91 ± 0.58 in Group I, *p* = 0.049).

### 3.3. Percutaneous Coronary Intervention-Related Findings

Table 3 demonstrates that before the PSM, femoral artery access was utilized in more Group II lesions than Group I (30.85% vs. 14.62%, with *p* = 0.002). Concerning the type of balloon utilized for pre-dilation, Group II had more patients using scoring balloons (3.19% vs. 0% in Group I with *p* = 0.019). In Group I, pre-dilation balloons were longer but had a lower maximum pressure than in Group II (17.51 ± 2.53 vs. 16.61 ± 2.68 with *p* = 0.008 and 11.62 ± 3.03 vs. 12.48 ± 2.93 with *p* = 0.029, respectively). Plaque modification was utilized by 4.26% of lesions in Group II, compared to 0% in Group I with *p* = 0.007. After the PSM, the utilization rate of femoral vascular access was greater in Group II (30.56%) than in Group I (14.81%) with *p* = 0.019. There were no differences in balloon type, length, or maximum pressure for pre-dilation balloons between the two groups following PSM.

In terms of stent information, prior to the PSM, Group I had greater stent diameter size (3.35 ± 0.51 vs. 3.21 ± 0.50, *p* = 0.034), stent-balloon maximum pressure (13.99 ± 2.84 vs. 13.18 ± 2.58, *p* = 0.022), and stent maximum diameter at deployment (3.42 ± 0.52 vs. 3.24 ± 0.52, *p* = 0.008), but Group I had shorter total stent length (26.00 (22.00,34.00) vs. 30.00 (22.00,38.00), *p* = 0.010) and fewer number of stents per lesion (1.07 ± 0.26 vs. 1.16 ± 0.37, *p* = 0.038). Post-dilation was performed on 77.66% of lesions in Group II, which is a significantly higher percentage than the 62.57% in Group I (*p* = 0.012). The percentage of lesions in Group II, which used an N.C. balloon for post-dilation (72.34%), was significantly higher than those in Group I (57.89%) with *p* = 0.020. The post-dilation balloon diameter size and the maximum diameter were found to be significantly higher in Group I (3.61 ± 0.53 vs. 3.39 ± 0.44, and 3.71 ± 0.52 vs. 3.48 ± 0.44, respectively, with *p* < 0.01 for both comparisons). Following the PSM, all of the parameters associated with the stent were comparable between the two groups. All post-dilation-related parameters were comparable in both groups, except that the lesions in Group II used post-dilation more frequently (80.56% vs. 65.43% in Group I, *p* = 0.036), and post-dilation balloon length in Group II was significantly longer (12.83 ± 3.82 vs. 11.42 ± 3.55 in Group I, *p* = 0.047).

### 3.4. IVUS Images Analysis

Table 4 shows all IVUS measurements pre- and post-intervention. Prior to the PSM, pre-intervention IVUS revealed that the minimum luminal area was smaller in Group II (2.44 ± 0.67 mm^2^ vs. 2.67 ± 0.90 mm^2^, *p* = 0.019). Group II also exhibited a more significant plaque burden (81.34% (78.45,85.29) vs. 79.83% (74.57,83.41), *p* = 0.006) and a longer lesion length (30.88 ± 11.09 vs. 26.76 ± 9.73, *p* = 0.002) than Group I. At the reference sites, Group I had a greater luminal area and a lower plaque burden than Group II. All calcium morphology-related measures, including maximum calcium arc, calcium length, calcium index, calcium nodule, super calcium, and deep calcium were greater in Group II than in Group I. After the PSM, both groups exhibited equivalent luminal area at the minimal luminal area site (2.69 ± 0.91 vs. 2.49 ± 0.69, *p* = 0.120) and the proximal reference (11.73 ± 4.68 vs. 10.85 ± 4.54, *p* = 0.244). Group II also had greater plaque burdens at the minimal luminal area site (81.43% (78.40,85.59) vs. 80.27% (73.67,84.00), *p* = 0.041), the proximal reference (45.15 ± 13.47 vs. 38.86 ± 12.82, *p* = 0.004), and the distal reference sites (35.89 ± 14.14 vs. 30.45 ± 11.14, *p* = 0.009) than Group I. Group II continued to have greater values for all morphological calcification criteria compared to Group I even after the PSM was applied.

Prior to the PSM, compared to Group I, the IVUS examination after PCI revealed that Group II had a smaller MSA (5.71 ± 2.04 vs. 6.68 ± 2.34, *p* = 0.001), minimum stent diameter (2.36 ± 0.44 vs. 2.58 ± 0.46, *p* = 0.001), and maximum stent diameter (2.96 ± 0.54 vs. 3.17 ± 0.56, *p* = 0.04). After PSM, only Group II’s minimum stent diameter remained smaller (2.39 ± 0.44 vs. 2.54 ± 0.43, *p* = 0.028).

Regarding the stent expansion rate, before the PSM, Group II had a smaller stent expansion rate based on the MSA/MVA at the MSA site (38.83 ± 8.89 vs. 45.42 ± 9.07, *p* < 0.001), absolute MSA > 5.5 mm^2^ (41.49% vs. 66.08%, *p* = 0.006), and ULTIMATE criteria (57.45% vs. 74.27%, *p* = 0.005). Recent criteria, such as conventional criteria or IVUS-XPL criteria, revealed no difference in the expansion rate of stents between the two groups. Following the PSM, while the percentage of lesions achieving the MSA/MVA at the MSA site and MSA > 5.5 mm^2^ criteria was still lower in Group II (40.41 ± 8.67 vs. 44.56 ± 9.44 with *p* = 0.006 and 45.83% vs. 65.43% with *p* = 0.015, respectively), the percentage of lesions achieving the other criteria was comparable between the two groups.

Before the PSM, the percentage of lesions with malapposition was greater in Group II (45.74% vs. 24.56%, *p* < 0.001). Still, the proportion of lesions with major malapposition did not significantly differ between the two groups (12.28% vs. 18.09%, *p* = 0.197). After the PSM, the percentage of lesions with major malapposition was not significantly different between the two groups (11.11% vs. 22.22%, *p* = 0.064). Other complications, including tissue protrusion and stent edge dissection, were comparable across the two groups both before and after the PSM.

### 3.5. Clinical Endpoints

The cardiac events that occurred in both Groups during the follow-up period are detailed in Table 5. The mean follow-up duration was 18.6 ± 5.18 months. Before the PSM, the MACE (composite of cardiac death, MI, and TLR) was 1.99% in Group I, which was not significantly different from Group II with 1.09% with *p* = 0.594. Secondary outcomes, including Patient-oriented composite endpoint, all-cause mortality or components of MACE (cardiac death, MI, and TLR), TVR, Stroke, and Stent thrombosis, were comparable between the two groups. After the PSM, the MACE rates in Group I were 0%, comparable with 1.41% in Group II with *p* = 0.317. The secondary endpoints remained not significantly different between the two groups.

## 4. Discussion

After exclusion, 243 patients with 265 lesions in the CAPIRO study population were evaluated. In our investigation, we categorized the patients into two groups, with Group II having moderate/severe calcification based on a maximum calcium arc >180° and calcium length >5 mm. Maximum calcium arc >180° and calcium length >5 mm were independent indicators of the stent under expansion and worse clinical outcomes based on intravascular imaging [13,28,33]. Regarding the baseline characteristics, there were substantial differences between the two groups in terms of age, male gender, diabetes mellitus, current smoking, and chronic kidney disease. We performed the PSM for these factors to eliminate the bias of baseline features since these characteristics could affect the rate of calcified lesions as well as cardiac events [7]. All of the baseline characteristics of the two groups were equivalent following the PSM.

In this prospective, multi-center study, the proportion of patients with moderate/severe calcification lesions was 37.86%. With IVUS-guided PCI, the major adverse cardiac events (MACE) rate was comparable between the two groups (1.99% vs. 1.09%, *p* = 0.594 before the PSM and 0% vs. 1.41%, *p* = 0.317 after the PSM) at a mean follow-up of 18.6 months. There was no difference in MACE between patients with moderate/severe calcification lesions and those with non/mild calcification lesions. Both groups had similar rates of MACE components such as cardiac death, stroke, MI, TLR, and TVR. These findings show that IVUS-guided PCI could improve intervention outcomes in patients with calcified lesions, which is not inferior to lesions without calcifications. Recent studies comparing IVUS-guided PCI to angiography-guided PCI have revealed favorable outcomes. Several observational studies and meta-analyses involving a greater number of patients indicated that IVUS-guided PCI in patients with complex lesions was linked with a decreased mortality rate and major adverse cardiac events [18,34]. The use of IVUS in PCI with CTO lesions also showed that patients with IVUS-guided post-stent optimization had a lower rate of TLR/reocclusion than patients without IVUS [19]. In 2023, a prospective, multi-center study showed that in PCI with complex lesions, imaging-guided PCI was associated with a lower incidence of target lesion failure, including cardiac death, myocardial infarction, and target vessel revascularization [35]. In 2019, the MACE-trial study, which is a prospective study, evaluated the impact of calcified lesions on the outcomes of PCI. According to this study’s findings, moderate calcification had similar outcomes compared with non/mild calcification; however, severe calcification still had a negative impact on PCI outcomes [6]. Therefore, using a new-generation stent system and new devices to prepare lesions before implanting the stent could improve the outcomes of PCI. The main results of our study showed that IVUS-guided PCI in calcified lesions could lead to better outcomes, which is comparable with the outcomes in patients with non-complex lesions treated with IVUS-guided PCI.

In order to minimize the impact of diverse stent systems on the outcomes, our study utilized a single type of second-generation DES for each patient. Second-generation DES was linked with a decreased rate of patient-oriented endpoint or target lesion failure in individuals with coronary artery calcification compared to first-generation DES [5]. However, the rate of target lesion failure and stent thrombosis was greater in patients with moderate or severe calcification lesions than in patients with non-calcified or mild calcification lesions [6,36]. In our study, the outcomes of patients with moderate/severe calcification were equivalent to those of patients with no/mild calcification, indicating that IVUS guidance with second-generation DES could improve PCI outcomes, even in calcified lesions.

Concerning the stent expansion rate, the MSA in Group I (6.68 ± 2.34 mm^2^) was larger than in Group II (5.71 ± 2.04 mm^2^) with *p* < 0.001; however, following the PSM, this difference (6.42 ± 2.10 vs. 5.89 ± 2.15) was not significant with *p* = 0.126. Group II had a smaller minimum stent diameter than Group I before and after PSM with *p* < 0.05. Comparing the stent expansion rate by recent expansion criteria, based on MSA > 5.5 mm^2^ and MSA/MVA at the MSA site criteria, Group I had more lesions that achieved these criteria than Group II. Fujimura et al. (2019) compared numerous stent expansion rate criteria. They suggested that compared to absolute MSA and other expansion indexes, the MSA/MVA at the MSA site could be independently associated with an increased rate of TLR and stent thrombosis after two years [25]. However, in our study, more than 30% of lesions had moderate to severe calcification, and the ultrasound could not penetrate the calcium; therefore, the measurement of MVA in calcified lesions by IVUS could be imprecise. Therefore, the MSA/MVA at the MSA site criterion was not appropriate for comparing the expansion rate in our investigation. Based on IXUS-XPL criteria, the number of lesions that achieved the criteria was comparable between both groups. In the IVUS-XPL trial, 54% of patients achieved the expansion criteria, and these patients had a significantly lower rate of MACE compared to patients who did not meet the criteria [23]. The proportion of lesions achieved IVUS-XPL in our study was lower than in the IVUS-XPL trial but comparable with results from the study of Fujimura et al. (2021) [25]. In our study, we analyzed all “all-comer” patients, so we think the ULTIMATE criteria should be suitable to compare the expansion rate. Based on the ULTIMATE criteria, before PSM, Group I had more lesions that achieved this criterion compared to Group II, but after PSM, there was no significant difference. Although the expansion rate based on different expansion criteria was heterogeneous, in our study, with the guidance of IVUS and the use of second-generation DES, the expansion rate and the outcome of patients with moderate/severe calcification were comparable with patients with non/mild calcification. Moreover, in our study, a significant proportion of procedures that use non-compliant balloons for optimizing the stent expansion contribute to a better stent expansion rate and clinical outcomes.

However, interpreting the results of our study should be concerned with these limitations: (1) Although the prospective, multi-center study, the CAPIRO study patient population was small; therefore, the number of cardiac events was still low compared to other randomized trials. (2) In our study, we included “all-comer” patients, including all ACS or non-ACS patients, with complex or non-complex lesions. Although this patient recruitment method is suitable for real clinical practice, the heterogeneity in baseline and lesion characteristics should be considered. (3) In 300 patients from the CAPIRO study, we excluded 50 patients who met the exclusion criteria for IVUS image analysis. The exclusion of a significant proportion of patients could have a considerable effect on the results of our study. (4) In patients with moderate/severe calcification, only about 4% were treated with plaque modification methods before implanting the stent. Therefore, in our study, we could not assess the role of plaque modification methods in the procedure outcomes. (5) Although we address the cardiac events for more than one-year follow-up, to assess the long-term benefits of IVUS-guided PCI, the follow-up time will be increased to 3 years.

## 5. Conclusions

Through this prospective, multi-center study, IVUS-guided PCI in calcified lesions was not inferior compared to PCI in non/mild calcified lesions. Although the expansion rate based on some criteria was lower in groups with moderate/severe calcification, other criteria showed that the expansion rate was comparable between both groups. The clinical outcomes after mid-term follow-up were similar between patients with moderate/severe calcification and patients with none/mild calcification. However, future study with a larger population and longer follow-up time is needed to confirm the role of IVUS-guided PCI in calcified lesions.

## Figures and Tables

**Table 1:** Baseline Clinical Characteristics.

	Non/Mild Calcification (n = 151)	Moderate/Severe Calcification (n = 92)	*p*	Matched Non/Mild Calcification (n = 71)	Matched Moderate/Severe Calcification (n = 71)	*p*
Baseline Characteristics						
Age (years)	63.60 ± 9.75	67.71 ± 9.77	0.002	66.48 ± 10.14	66.96 ± 9.05	0.767
Age > 65 years old	40.40% (61)	61.96% (57)	0.001	49.30% (35)	59.15% (42)	0.238
Male	82.12% (124)	65.22% (60)	0.003	76.06% (54)	73.24% (52)	0.700
BMI (g/m^2^)	24.96 ± 3.19	25.25 ± 3.11	0.498	24.78 ± 3.44	25.23 ± 2.87	0.393
Hypertension	69.54% (105)	80.43% (74)	0.061	76.06% (54)	76.06% (54)	1
Diabetes Mellitus	24.50% (37)	53.26% (49)	0.001	29.58% (21)	45.07% (32)	0.056
Current Smoking	29.14% (44)	14.13% (13)	0.035	18.31% (13)	16.90% (12)	0.714
Chronic Kidney Disease	1.32% (2)	8.70% (8)	0.005	2.82% (2)	4.23% (3)	0.649
Clinical Presentation			0.176			0.647
STEMI NSTEMI UAP Non-ACS	1.99% (3) 11.92% (18) 54.30% (82) 31.79% (48)	2.17% (2) 20.65% (19) 55.43% (51) 21.74% (20)	0.921 0.066 0.864 0.091	4.23% (3) 14.08% (10) 50.70% (36) 30.99% (22)	2.82% (2) 15.49% (11) 59.15% (42) 22.54% (16)	0.649 0.813 0.312 0.255
Multi-lesions PCI	7.28% (11)	10.87% (10)	0.335	5.63% (4)	8.45% (6)	0.512
Family history of CAD	5.96% (9)	2.17% (2)	0.221	2.82% (2)	2.82% (2)	0.508
Previous PCI	5.96% (9)	13.04% (12)	0.057	4.23% (3)	8.45% (6)	0.301
TIA	8.61% (13)	9.78% (9)	0.757	7.04% (5)	8.45% (6)	0.754
Laboratory Findings						
Hemoglobin (10^3^/μL)	14.10 (13.10,15.00)	13.15 (12.00,14.60)	<0.001	13.80 (12.70,14.70)	13.30 (12.00,14.60)	0.257
Creatinine (mg/dL)	0.88 (0.74,1.01)	0.88 (0.73,1.17)	0.295	0.87 (0.71,1.06)	0.87 (0.73,1.17)	0.485
EGFR (mL/min/m^2^)	85.80 ± 22.55	76.66 ± 31.38	0.016	82.95 ± 25.68	79.35 ± 28.86	0.434
EGFR < 60 (mL/min/m^2^)	7.95% (12)	25.00% (23)	<0.001	11.27% (8)	22.54% (16)	0.073
Dyslipidemia *	58.28% (88)	71.74% (66)	0.035	66.20% (47)	71.83% (51)	0.468
LVEF (%)	61.00 (58.00,66.00)	58.00 (55.00,62.00)	0.001	60.00 (57.00,64.00)	58.00 (56.00,63.00)	0.108
HbA1C (%)	5.80 (5.40,6.40)	6.30 (5.78,7.50)	<0.001	5.80 (5.50,6.30)	6.15 (5.70,7.43)	0.011
Pre-PCI Medications						
Aspirin Betablocker RAS Statin P2Y12	22.52% (34) 19.21% (29) 36.42% (55) 38.41% (58) 84.11% (127)	29.35% (27) 26.09% (24) 52.17% (48) 59.78% (55) 69.57% (64)	0.234 0.187 0.020 0.001 0.007	26.76% (19) 22.54% (16) 38.03% (27) 49.30% (35) 81.69% (58)	30.99% (22) 19.72% (14) 52.11% (37) 59.15% (42) 74.65% (53)	0.579 0.565 0.127 0.238 0.310

Abbreviations: BMI = Body Mass Index; STEMI = ST-Elevation Myocardial Infarction; NSTEMI = Non-ST-Elevation Myocardial Infarction; UAP = Unstable Angina; Non-ACS = Non-Acute Coronary Syndrome; PCI = Percutaneous Coronary Intervention; CAD = Coronary Artery Disease; TIA = Transient Ischemic Attacks; EGFR = Estimates Glomerular Filtration Rate, based on MDRD equation; LVEF = Left Ventricular Ejection Fraction. * Dyslipidemia: LDL cholesterol more than 140 mg/dl or Total cholesterol more than 220 mg/dl or treated with medications.

**Table 2:** Coronary Angiography Findings.

	Non/Mild Calcification (n = 171)	Moderate/Severe Calcification (n = 94)	p	Matched Non/Mild Calcification (n = 81)	Matched Moderate/Severe Calcification (n = 72)	*p*
Artery			0.879			0.616
LAD	68.42% (117)	70.21% (66)	0.763	67.90% (55)	75.00% (54)	0.333
LCX	11.11% (19)	11.70% (11)	0.884	9.88% (8)	8.33% (6)	0.741
RCA	19.30% (33)	18.09% (17)	0.809	22.22% (18)	16.67% (12)	0.388
Left Main	0.58% (1)	0% (0)	0.458	0% (0)	0% (0)	N/A
Ramus	0.58% (1)	0% (0)	0.458	0% (0)	0% (0)	N/A
AHA/ACC Classification			0.010			0.374
A	1.17% (2)	0% (0)	0.293	1.23% (1)	0% (0)	0.344
B1	14.04% (24)	5.32% (5)	0.030	12.35% (10)	5.56% (4)	0.146
B2	49.12% (84)	40.43% (38)	0.174	41.98% (34)	44.44% (32)	0.758
C	35.67% (61)	54.26% (51)	0.003	44.44% (36)	50.0% (36)	0.492
TIMI Grade			0.091			0.398
TIMI-0	4.68% (8)	2.13% (2)	0.297	4.94% (4)	2.78% (2)	0.492
TIMI-1	11.11% (19)	10.64% (10)	0.906	7.41% (6)	12.50% (9)	0.290
TIMI-2	21.05% (36)	10.64% (10)	0.032	18.52% (15)	11.11% (8)	0.201
TIMI-3	63.16% (108)	76.60% (72)	0.025	69.14% (56)	73.61% (53)	0.542
Total Occlusion	4.09% (7)	6.38% (6)	0.409	4.94% (4)	8.33% (6)	0.396
Chronic total occlusion	3.51% (6)	5.32% (5)	0.480	3.70% (3)	6.94% (5)	0.369
Ostium Lesion	1.75% (3)	2.13% (2)	0.831	2.47% (2)	2.78% (2)	0.905
QCA Analysis						
Lesion Diameter (mm)	1.03 ± 0.38	0.95 ± 0.37	0.094	0.97 ± 0.37	0.94 ± 0.38	0.611
Lesion Reference Diameter (mm)	2.94 ± 0.59	2.67 ± 0.63	0.001	2.91 ± 0.58	2.70 ± 0.63	0.049
Diameter Stenosis (%)	66.23 ± 12.76	66.85 ± 14.02	0.718	67.66 ± 13.96	68.17 ± 14.86	0.828
Lesion Area (mm^2^)	0.82 (0.43,1.24)	0.70 (0.35,1.13)	0.157	0.74 (0.38,1.11)	0.62 (0.32,1.16)	0.603
Lesion Reference Area (mm^2^)	6.45 (5.20,8.49)	5.50 (3.98,7.23)	0.001	6.27 (5.20,7.37)	5.70 (3.88,7.26)	0.077
Area Stenosis (%)	87.98 ± 7.76	87.07 ± 7.97	0.931	87.61 ± 8.20	87.69 ± 8.28	0.956
Lesion Length (mm)	23.95 ± 8.56	27.30 ± 9.94	0.005	24.83 ± 9.12	27.16 ± 9.18	0.132
Proximal Mean Diameter (mm)	3.28 ± 0.56	3.04 ± 0.60	0.002	3.25 ± 0.54	3.08 ± 0.61	0.072
Lesion Mean Diameter (mm)	2.30 ± 0.47	2.10 ± 0.44	0.001	2.24 ± 0.43	2.14 ± 0.44	0.160
Distal Mean Diameter (mm)	2.92 ± 0.64	2.75 ± 0.69	0.047	2.89 ± 0.64	2.76 ± 0.72	0.257
Proximal Mean Area (mm^2^)	3.27 ± 0.65	3.10 ± 0.64	0.049	3.28 ± 0.58	3.15 ± 0.66	0.212
Lesion Mean Area (mm^2^)	2.31 ± 0.49	2.12 ± 0.45	0.003	2.27 ± 0.45	2.16 ± 0.45	0.178
Distal Mean Area (mm^2^)	2.95 ± 0.77	2.84 ± 0.84	0.288	2.99 ± 0.89	2.89 ± 0.90	0.503
Plaque Symmetry	0.70 (0.49,0.87)	0.67 (0.56,0.82)	0.856	0.76 (0.50,0.87)	0.65 (0.56,0.81)	0.211
Plaque Area (mm^2^)	13.44 (9.95,18.75)	13.53 (10.25,18.65)	0.766	13.79 (9.77,20.15)	12.97 (10.35,18.14)	0.592

Abbreviations: LAD = Left Anterior Descending, LCX = Left Circumflex, RCA = Right Coronary Artery, AHA/ACC = American Heart Association/American College of Cardiology, TIMI = Thrombolysis In Myocardial Infarction, QCA = Quantitative Coronary Angiography.

**Table 3:** PCI-related findings.

	Non/Mild Calcification (n = 171)	Moderate/ Severe Calcification (n = 94)	*p*	Matched Non/Mild Calcification (n = 81)	Matched Moderate/Severe Calcification (n = 72)	*p*
Vascular Access			0.002			0.019
Radial	85.38% (146)	69.15% (65)		85.19% (69)	69.44% (50)	
Femoral	14.62% (25)	30.85% (29)		14.81% (12)	30.56% (22)	
Pre-dilation	97.08% (166)	97.87% (92)	0.699	98.77% (80)	98.61% (71)	0.933
Pre-Stent-Balloon type			0.103			0.269
Semi-compliant balloon	94.74% (162)	90.43% (85)	0.182	97.53% (79)	90.28% (65)	0.057
Non-compliant balloon	2.34% (4)	3.19% (3)	0.679	1.23% (1)	2.78% (2)	0.492
Stent-balloon	0% (0)	1.06% (1)	0.177	0% (0)	1.39% (1)	0.287
Scoring balloon	0% (0)	3.19% (3)	0.019	0% (0)	4.17% (3)	0.064
Pre-dilation balloon diameter size (mm)	2.57 ± 0.37	2.64 ± 0.38	0.208	2.58 ± 0.35	2.62 ± 0.39	0.457
Pre-dilation balloon length (mm)	17.51 ± 2.53	16.61 ± 2.68	0.008	17.24 ± 2.52	17.01 ± 2.88	0.612
Pre-dilation balloon maximum pressure (atm)	11.62 ± 3.03	12.48 ± 2.93	0.029	11.96 ± 3.12	12.28 ± 3.11	0.531
Pre-dilation balloon maximum diameter (mm)	2.68 ± 0.37	2.76 ± 0.38	0.101	2.69 ± 0.32	2.74 ± 0.39	0.420
Plaque modification	0% (0)	4.26% (4)	0.007	0% (0)	4.17% (3)	0.064
Rotablator	0% (0)	1.06% (1)	0.177	0% (0)	0% (0)	N/A
Scoring balloon	0% (0)	3.19% (3)	0.019	0% (0)	4.17% (3)	0.064
Stent diameter size (mm) *	3.35 ± 0.51	3.21 ± 0.50	0.034	3.28 ± 0.47	3.25 ± 0.53	0.673
Stent length size (mm) *	26.00 (22.00,34.00)	30.00 (22.00,34.00)	0.062	26.00 (22.00,34.00)	30.00 (26.00,37.00)	0.113
Number of stents per lesion	1.07 ± 0.26	1.16 ± 0.37	0.038	1.09 ± 0.28	1.14 ± 0.35	0.306
Total stent length (mm)	26.00 (22.00,34.00)	30.00 (22.00,38.00)	0.010	26.00 (22.00,34.00)	30.00 (26.00,38.00)	0.062
Stent-balloon maximum pressure at deployment (atm) *	13.99 ± 2.84	13.18 ± 2.58	0.022	13.62 ± 2.85	13.35 ± 2.77	0.554
Stent maximum diameter at deployment (mm) *	3.42 ± 0.52	3.24 ± 0.52	0.008	3.34 ± 0.47	3.28 ± 0.56	0.487
Post-dilation	62.57% (107)	77.66% (73)	0.012	65.43% (53)	80.56% (58)	0.036
Post-dilation balloon type			0.072			0.151
Semi-compliant balloon	1.75% (3)	3.19% (3)	0.452	1.23% (1)	1.39% (1)	0.933
Non-compliant balloon	57.89% (99)	72.34% (68)	0.020	64.20% (52)	77.78% (56)	0.066
Stent-balloon	2.92% (5)	2.13% (2)	0.699	0% (0)	1.39% (1)	0.287
Post-dilation balloon diameter size (mm)	3.61 ± 0.53	3.39 ± 0.44	0.005	3.58 ± 0.51	3.43 ± 0.44	0.100
Post-dilation balloon length (mm)	12.32 ± 3.92	12.84 ± 3.76	0.377	11.42 ± 3.55	12.83 ± 3.82	0.047
Post-dilation balloon maximum pressure (atm)	16.70 ± 3.67	16.38 ± 3.31	0.554	16.72 ± 4.00	16.34 ± 2.93	0.575
Post-dilation balloon maximum diameter (mm)	3.71 ± 0.52	3.48 ± 0.44	0.003	3.69 ± 0.50	3.52 ± 0.45	0.072

* If a lesion was implanted with multiple stents, stent diameter size, stent length size, stent maximum pressure at deployment, and stent maximum diameter at deployment were derived from the parameters of the stent with the largest diameter size.

**Table 4:** IVUS findings.

	Non/Mild Calcification (n = 171)	Moderate/Severe Calcification (n = 94)	*p*	Matched Non/Mild Calcification (n = 81)	Matched Moderate/Severe Calcification (n = 72)	*p*
Pre-PCI IVUS						
Minimum luminal area site						
Luminal Area (mm^2^)	2.67 ± 0.90	2.44 ± 0.67	0.019	2.69 ± 0.91	2.49 ± 0.69	0.120
Vessel Area (mm^2^)	13.56 ± 4.92	13.74 ± 4.59	0.772	13.94 ± 4.65	14.23 ± 4.80	0.702
Plaque Burden (%)	79.83 (74.57,83.41)	81.34 (78.45,85.29)	0.006	80.27 (73.67,84.00)	81.43 (78.40,85.59)	0.041
Remodeling Index	0.86 ± 0.23	0.89 ± 0.21	0.294	0.88 ± 0.22	0.90 ± 0.21	0.565
Lesion Length (mm)	26.76 ± 9.73	30.88 ± 11.09	0.002	27.88 ± 9.44	31.56 ± 11.27	0.029
Proximal reference site Luminal Area (mm^2^) Vessel Area (mm^2^) Plaque Burden (%)	11.82 ± 4.53 18.75 ± 5.7 36.98 ± 12.40	10.41 ± 4.23 18.76 ± 5.35 44.78 ± 12.98	0.014 0.985 0.001	11.73 ± 4.68 19.23 ± 6.19 38.86 ± 12.82	10.85 ± 4.54 19.49 ± 5.29 45.15 ± 13.47	0.244 0.780 0.004
Distal reference site Luminal Are (mm^2^) Vessel Area (mm^2^) Plaque burden (%)	8.21 (6.27,11.03) 13.20 ± 5.70 30.46 ± 10.97	7.04 (5.33,10.18) 12.64 ± 5.43 35.05 ± 14.46	0.012 0.435 0.004	8.15 (6.41,10.62) 13.18 ± 5.42 30.45 ± 11.14	7.04 (5.18,10.38) 12.83 ± 5.70 35.89 ± 14.14	0.034 0.702 0.009
Mean Reference Luminal Area (mm^2^)	10.45 ± 3.88	9.19 ± 3.49	0.010	10.40 ± 3.92	9.43 ± 3.71	0.119
Volumetric analysis						
Mean luminal area (mm^3^/mm)	5.89 (4.70,7.68)	5.04 (4.00,5.96)	<0.001	5.82 (4.77,7.27)	5.16 (4.04,6.26)	0.028
Mean vessel area (mm^3^/mm)	14.98 ± 4.98	14.87 ± 4.35	0.856	14.97 ± 4.47	15.39 ± 4.48	0.567
Plaque burden (%)	56.80 ± 8.44	63.47 ± 7.28	<0.001	57.56 ± 9.28	63.91 ± 7.25	<0.001
Maximum calcium arc (^o^)	86.70 (43.90,139.20)	266.80 (226.28,360.00)	<0.001	96.30 (67.20,148.05)	263.95 (222.58,360.00)	<0.001
Calcium length (mm)	6.10 (2.10,13.10)	20.30 (14.23,26.55)	<0.001	7.50 (3.30,12.75)	21.65 (15.20,26.50)	<0.001
Calcium Index *	10.06 ± 11.22	56.63 ± 24.24	<0.001	11.61 ± 12.23	55.90 ± 23.34	<0.001
Superficial Calcium	80.12% (137)	100.0% (94)	<0.001	88.89% (72)	100.0% (72)	0.004
Deep Calcium	7.60% (13)	26.60% (25)	<0.001	7.41% (6)	25.00% (18)	0.003
Calcium nodule	6.43% (11)	23.40% (22)	<0.001	9.88% (8)	25.00% (18)	0.013
Plaque rupture	15.20% (26)	39.36% (37)	<0.001	18.52% (15)	45.83% (33)	<0.001
Attenuate Plaque	71.35% (122)	79.79% (75)	0.132	74.07% (60)	79.17% (57)	0.459
POST-PCI IVUS						
Minimum stent area site						
Minimum stent area (mm^2^)	6.68 ± 2.34	5.71 ± 2.04	0.001	6.42 ± 2.10	5.89 ± 2.15	0.126
Vessel area at MSA (mm^2^)	15.02 ± 5.07	14.71 ± 5.06	0.643	14.83 ± 4.91	14.95 ± 5.30	0.891
Maximum stent diameter (mm)	3.17 ± 0.56	2.96 ± 0.54	0.04	3.11 ± 0.51	3.02 ± 0.56	0.286
Minimum stent diameter (mm)	2.58 ± 0.46	2.36 ± 0.44	0.001	2.54 ± 0.43	2.39 ± 0.44	0.028
Stent asymmetry	0.17 (0.12,0.23)	0.17 (0.13,0.25)	0.248	0.17 (0.12,0.22)	0.19 (0.14,0.26)	0.060
Stent eccentricity	0.83 (0.77,0.88)	0.83 (0.75,0.87)	0.248	0.83 (0.78,0.88)	0.81 (0.74,0.86)	0.060
Mean stent area (mm^2^)	8.16 (6.63,11.07)	7.39 (5.87,9.85)	0.055	7.70 (6.47,11.21)	8.22 (5.94,10.33)	0.642
Conventional stent expansion	65.91 ± 14.71	64.38 ± 13.86	0.127	63.94 ± 13.83	65.11 ± 14.19	0.607
MSA/MVA at MSA site (%)	45.42 ± 9.07	38.83 ± 8.89	<0.001	44.56 ± 9.44	40.41 ± 8.67	0.006
IVUS-XPL * trial stent expansion criteria	11.70% (20)	11.70% (11)	0.999	6.17% (5)	13.89% (10)	0.109
ULTIMATE * trial stent expansion criteria	74.27% (127)	57.45% (54)	0.005	70.37% (57)	63.89% (46)	0.394
MSA > 5.5 mm^2^	66.08% (113)	41.49% (39)	0.006	65.43% (53)	45.83% (33)	0.015
MSA/Average reference lumen area > 80%	17.54% (30)	13.83% (13)	0.433	13.58% (11)	15.28% (11)	0.765
Stent malapposition	24.56% (42)	45.74% (43)	<0.001	24.69% (20)	52.78% (38)	<0.001
Major Stent Malapposition	12.28% (21)	18.09% (17)	0.197	11.11% (9)	22.22% (16)	0.064
Minor Stent Malapposition	12.28% (21)	27.66% (26)	0.002	13.58% (11)	30.56% (22)	0.011
Tissue protrusion	1.75% (3)	1.06% (1)	0.659	2.47% (2)	1.39% (1)	0.630
Stent Edge Dissection	1.17% (2)	1.06% (1)	0.938	1.23% (1)	1.39% (1)	0.933

Abbreviations: MSA = Minimum Stent Area, ULTIMATE criteria = MSA > 5.5 mm^2^ or >90% distal lumen reference area, IVUS-XPL criteria = MSA > distal lumen reference area, Conventional stent expansion = MSA/(Average reference lumen area) × 100, Calcium Index = (maximum calcium arc/360) × (total calcium length/lesion length) × 100, Major Stent Malapposition: axial distance > 0.4 mm or >1 mm in length.

**Table 5:** MACE after one-year follow-up.

	Non/Mild Calcification (n = 151)	Moderate/Severe Calcification (n = 92)	*p*	Matched Non/Mild Calcification (n = 71)	Matched Moderate/Severe Calcification (n = 71)	*p*
MACE	1.99% (3)	1.09% (1)	0.594	0% (0)	1.41% (1)	0.317
Cardiac Death	0.66% (1)	0% (0)	0.435	0% (0)	0% (0)	N/A
MI	0% (0)	0% (0)	N/A	0% (0)	0% (0)	N/A
TLR	1.32% (2)	1.09% (1)	0.870	0% (0)	1.41% (1)	0.317
POCE	5.96% (9)	3.26% (3)	0.303	7.04% (5)	2.82% (2)	0.244
All-cause Death	4.64% (7)	2.17% (2)	0.276	7.04% (5)	1.41% (1)	0.092
Stroke	1.32% (2)	0% (0)	0.269	1.41% (1)	0% (0)	0.317
TVR	1.32% (2)	0% (0)	0.266	0% (0)	0% (0)	N/A
OVR	1.99% (3)	4.35% (4)	0.343	4.23% (3)	4.23% (3)	0.965
CHF	0.66% (1)	1.09% (1)	0.723	0% (0)	1.41% (1)	0.317
Stent Thrombosis	0% (0)	0% (0)	N/A	0% (0)	0% (0)	N/A

Abbreviations: MI = Myocardial Infarction, TLR = Target Lesion Revascularization, TVR = Target Vessel Revascularization, OVR = Other Vessel Revascularization, CHF = Chronic Heart Failure, MACE = Cardiac death or MI or TLR, POCE = All-cause death or MI or TLR or TVR.

## Data Availability

This article presents most of the study’s data in tables and figures. Additional data related to this study are available upon request from the corresponding author.
